# Incidence and Types of Fetal Chromosomal Abnormalities in First Trimester of Thai Pregnant Women between Miscarriages and Intrauterine Survivals

**DOI:** 10.1159/000527977

**Published:** 2023-03-01

**Authors:** Rachanee Parinayok, Prapatsorn Areesirisuk, Takol Chareonsirisuthigul, Warakorn Buchachat, Budsaba Rerkamnuaychoke

**Affiliations:** Human Genetic Laboratory, Department of Pathology, Faculty of Medicine, Ramathibodi Hospital, Mahidol University, Bangkok, Thailand

**Keywords:** Fetal chromosomal abnormality, First trimester, Miscarriage, Intrauterine survival, Maternal age

## Abstract

Abortion is a common pregnancy complication. Fetuses with several types of chromosomal abnormalities are aborted during the first trimester, while others have a better chance of surviving. This research aims to study and compare the incidence and types of fetal chromosomal abnormalities during the first trimester of Thai pregnant women between miscarriages and intrauterine survivals. Cytogenetic and BACs-on-Beads™ assays were assessed from 2010 to 2020 in Ramathibodi Hospital using first trimester samples of 265 chorionic villi as a retrospective study. Chromosomal abnormalities were observed in 135 cases (50.94%) including 38.11% miscarriages and 12.83% intrauterine survivals. In total, 75.56% single autosomal trisomies, 18.52% sex chromosome aneuploidies, 5.19% double aneuploidies, and 0.74% structural abnormalities were detected. In miscarriages, all chromosomes were involved in abnormalities except chromosomes 1, 5, 8, 9, 11, and 17, while survivals had only trisomy 13, 18, 21, and sex chromosome aneuploidy. Trisomy 16 and 18 were the most common abnormalities in miscarriages and intrauterine survivals, respectively. The highest rate of chromosomal aberrations was demonstrated in 8–9<sup>+6</sup> and 12–13<sup>+6</sup> weeks of gestation in miscarriages and intrauterine survivals, respectively. Correlation between chromosomal abnormalities and maternal age <35 years and ≥35 years was significant (*p* < 0.05) in intrauterine survival and first trimester groups.

## Introduction

Chromosome aberrations resulting from errors that occur during embryonic division and growth are an important factor in miscarriage within the first trimester [Hassold et al., 2007; Thomas et al., 2021]. Correlation between chromosomal abnormalities and the occurrence of miscarriage has been observed since the 1960s [Bowen et al., 1969]. Cytogenetic techniques (conventional karyotyping) and many molecular assays have been increasingly applied to investigate chromosomal abnormalities in miscarriages [Grati et al., 2015; Sahoo et al., 2017; Teles et al., 2017]. Several studies determined that 50–60% of first-trimester miscarriages were caused by fetal chromosomal abnormalities [Hassold et al., 1980; Eiben et al., 1990; Soler et al., 2017; Pylyp et al., 2018]. In Thailand, a study of fetal chromosomal abnormalities in pregnant women was performed during the second trimester using amniotic fluid samples with incidence rates of 2.5–3.3%. Abnormalities were trisomies 13, 18, 21, sex chromosome aneuploidies, and structural rearrangements [Kongyon and Puangsricharern, 2003; Ratanasiri et al., 2011; Pathompanitrat et al., 2013; Rawangkan et al., 2015]. The present study examined the incidence and types of fetal chromosomal abnormalities during the first trimester of Thai pregnant women. Data were compared between miscarriages and intrauterine survivals. No literature is available on chromosomal abnormalities during the first trimester of pregnancy, especially for first trimester miscarriage of Thai pregnant women. Many reports are available on other populations but the Thai population differs in environmental exposure and maternal health conditions from other countries. Accurate identification of genetic characteristics can provide important information for medical management, reproductive counseling, and supportive patient care.

## Materials and Methods

### Prenatal Specimens

This retrospective study was carried out by assessing cytogenetic analyses and the BACs-on-Beads^TM^ (BoBs^TM^) assay performed in the Human Genetic Laboratory, Department of Pathology, Faculty of Medicine Ramathibodi Hospital, Mahidol University between 2010 and 2020.

**Fig. 1:**
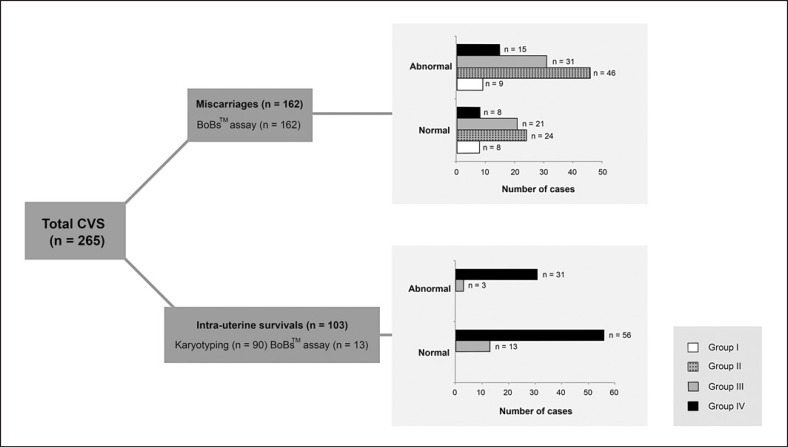
Workflow from 265 sampling processes for data evaluation. Group I, 5–7^+6^ weeks; Group II, 8–9^+6^ weeks; Group III, 10–11^+6^ weeks; Group IV, 12–13^+6^ weeks.

A total of 265 chorionic villus samples from first trimester pregnancies were analyzed, consisting of 162 samples from pregnant women who delivered dead embryos or fetuses, in which BoBs assay was performed. The remaining 103 chorionic villus samples were from pregnant women with a living fetus in utero; 90 samples were analyzed by the cytogenetic technique, while the other 13 were subjected to the BoBs assay (Fig. 1). The Ethical Clearance Committee, Faculty of Medicine, Ramathibodi Hospital, Mahidol University approved this study on human rights related to research involving human subjects (COA. MURA2021/811).

### Cytogenetic Technique

The cytogenetic technique using chorionic villus samples was modified from the AGT cytogenetic laboratory manual [Arsham et al., 2016]. At least 10 mg of the aspirated villus samples from first trimester pregnancies were carefully washed and estimated under an inverted microscope. Villus fragments were isolated from maternal tissues and blood clots and then disaggregated by mechanical and enzymatic methods. Mesenchymal cells of the villus core were released, and the fibroblasts were actively proliferative in a sterile 25-cm^2^ cell culture flask. Three independent cell cultures were performed. After 9–14 days, metaphase chromosomes were prepared for analysis. Karyotyping was performed by G-banding using the trypsin-Giemsa staining technique. Detailed chromosome analyses of 10 metaphase cells of the 400–550 bands per haploid set level were karyotyped and an additional 15 metaphase cells were examined. The aberrations and karyotypes were classified according to the International System for Human Cytogenomic Nomenclature 2020 (ISCN, 2020) [McGowan-Jordan et al., 2020].

### BACs-On-Beads^TM^ Assay

A KaryoLite BoBs kit was used to evaluate arm-specific aneuploidies in all 24 chromosomes in a single assay, according to the manufacturer's instructions (Wallac Oy PerkinElmer, Turku, Finland). Native villus fragments that excluded maternal tissue contamination were selected under an inverted microscope and directly performed for DNA extraction. A total of 50–250 ng genomic DNA was labeled with enzyme-linked biotin-deoxynucleoside triphosphate. The labeled products were purified and hybridized with normal DNA from reference males and females with BoBs probes and subjected to fluorescent hybridization incubation. Fluorescence signals were measured using a Luminex 200 platform, and the results were analyzed by BoBsoft 1.0 software (PerkinElmer) [Jaranasaksakul et al., 2015].

### Statistical Analysis

Incidence and types of fetal chromosomal abnormalities were compared between miscarriages and intrauterine survivals. Correlations between frequency of chromosomal aberrations and maternal age groups were analyzed by a χ^2^ test using SPSS18.0 software (SPSS Inc., Chicago, IL, USA). Statistical significance was determined at *p* < 0.05.

## Results

### Chromosomal Abnormalities and Weeks of Gestation

**Fig. 2:**
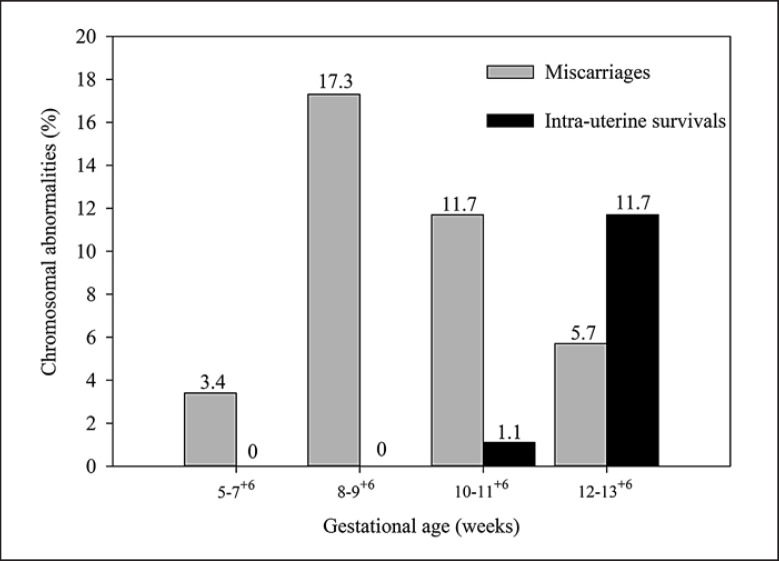
Incidence of total chromosomal abnormalities according to gestational age in miscarriages and intrauterine survivals.

Gestational ages were established according to ultrasonography. The 265 cases were classified into 4 groups (Group I: 5–7^+6^ weeks, Group II: 8–9^+6^ weeks, Group III: 10–11^+6^ weeks, and Group IV: 12–13^+6^ weeks) (Fig. 1; Table 1). Incidences of chromosomal abnormalities during first trimester pregnancies comprised 135 cases (50.94%) with 101 cases of miscarriage (38.11%) including 3.4%, 17.3%, 11.7%, and 5.7% in Groups I–IV, respectively. In 34 cases of intrauterine survivals (12.8%), chromosomal abnormalities were detected in 1.1% of Group III and 11.7% of Group IV (Fig. 2). The highest rate of chromosomal abnormalities was found in the miscarriage group, with 46 cases of 8–9^+6^ gestational weeks (28.4% of miscarriages), whereas intrauterine survival was observed in 31 cases of 12–13^+6^ gestational weeks (30.1% of intrauterine survival). Frequency of chromosomal abnormalities in each gestational group of miscarriages was approximately 1:2. By contrast, frequency of intrauterine survivals was 1:5 and 1:3 in Groups III and IV, respectively.

### Chromosomal Abnormalities and Maternal Age

**Fig. 3:**
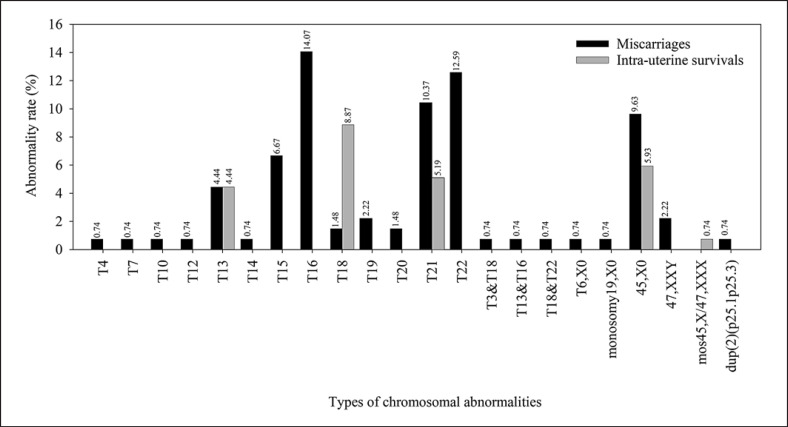
Incidence of chromosomal abnormalities in miscarriages and intrauterine survivals.

Incidence and type of chromosomal abnormalities compared between miscarriages and intrauterine survivals are shown in Table 1 and Figure 3, with incidence of main chromosomal abnormalities summarized in Table 2. Fetal chromosomal abnormalities were detected in 135 cases as 102 single autosomal trisomy cases (75.56%), 25 sex chromosomal aneuploidy cases (18.52%), 7 double aneuploidies (5.19%), and 1 structural abnormality case (0.7%). For miscarriages, chromosomal abnormalities were single autosomal trisomy (57.04%), double aneuploidy (5.19%), sex chromosomal aneuploidy (11.85%), and structural abnormality (0.74%), while in intrauterine survivals only single autosomal trisomy (18.52%) and sex chromosomal aneuploidy (6.67%) were observed.

Regarding the 162 miscarriages, fetal chromosomal abnormalities were detected in 101 cases (62.35%). Single autosomal trisomy was most frequent with 77 cases (47.53%); 16 cases (9.88%) showed sex chromosome aneuploidy. Seven cases (4.32%) were double aneuploidy, including 3 cases of double trisomy and 4 cases of combined abnormalities. One case (0.62%) was recorded for structural abnormality. Details of individual chromosomal abnormalities are shown in online supplementary Table 1 (see www.karger.com/doi/10.1159/000527977). Trisomies, including single trisomy, double trisomy and combined abnormalities, were found in all chromosomes except for chromosomes 1, 5, 8, 9, 11, and 17. The most common chromosomal abnormality was trisomy 16, followed by trisomy 22, trisomy 21, monosomy X and others, as shown in online supplementary Table 1.

Thirty-four out of 103 cases (33.01%) of intrauterine survivals had chromosomal abnormalities. Only trisomy 13, 18, 21, and sex chromosome aneuploidy were recorded, with 25 (24.27%) single autosomal trisomies being the most frequent, followed by sex chromosomal aneuploidy in 9 cases (8.74%). Trisomy 18 was the most common chromosomal abnormality, followed by monosomy X, trisomy 21, trisomy 13, and mos 45,X/47,XXX, respectively.

Maternal age ranged from 21 to 47 years in miscarriages and 13–45 years in intrauterine survivals. Chromosomal abnormalities by maternal age are shown in Table 3. The frequency of total chromosomal abnormalities in first trimester pregnancies and intrauterine survivals showed a statistically significant correlation with maternal age ≥35 years but it was not significant in miscarriages (*p* < 0.05) (Table 4).

## Discussion

Maternal age-related risk of a fetus with chromosomal abnormality remains a problem in maternal-fetal medicine. Nowadays, fetal chromosomal assessment is conducted by both noninvasive and invasive methods. Noninvasive methods include maternal factors and history, fetal ultrasound imaging, and maternal serum analyte or cell-free fetal DNA screening. The most popular method of noninvasive prenatal screening for chromosomal aneuploidy involves using circulating cell-free fetal DNA in maternal plasma. Because cell-free fetal DNA is mainly of placental trophoblastic origin, false positive and false negative results are possible. Moreover, at the early gestational age in which the test is performed, the quantity of circulating fetal DNA is very low, increasing the possibility of false negative results. However, invasive diagnostic methods performed by CVS or amniocentesis can provide definitive results. Chromosomal abnormalities are the most frequent fetal assessment problem during the first trimester of pregnant women. Chorionic villus sampling can be performed much earlier in pregnancy than amniocentesis. When both NIPT/CVS tests showed mosaicism results, amniocentesis was performed to follow up on the definitive survivor karyotypes. The traditional cytogenetic assay offers advantages of detection of additional fetal chromosomal abnormalities and some particular types of chromosomal abnormalities not identifiable through cell-free fetal DNA-based methods. Standard routine prenatal testing of noninvasive methods involves screening for common aneuploidies including trisomy 21, trisomy 18, trisomy 13, and sex chromosome abnormalities. Several laboratory methods have been developed to screen the most common chromosome aneuploidies but the cytogenetic assay is still the gold standard. Thailand is a middle-income country and faces economic problems in accessing prenatal genetic services of cell-free fetal DNA-based methods. Therefore, cytogenetic analysis is required to reduce the burden of genetic disorders and congenital disabilities that cause significant postnatal functional impairment.

The incidence of chromosomal abnormalities during first trimester miscarriages in previously published studies ranged considerably from 40 to 70% [Hassold et al., 1980; Eiben et al., 1990; Ljunger et al., 2005; Soler et al., 2017; Pylyp et al., 2018; Gu et al., 2021]. The types of cytogenetic alteration included single autosomal trisomy, sex chromosome aneuploidy, double aneuploidy, polyploidy, structural abnormality, and placental mosaicism. The correlation between chromosomal abnormalities and maternal age ≥35 years was significant [Hassold et al., 1980; Eiben et al., 1990; Ljunger et al., 2005; Soler et al., 2017; Pylyp et al., 2018; Gu et al., 2021].

However, in this study, fetal chromosomal abnormalities were detected in 62.35% of miscarriages. Single autosomal trisomy, double aneuploidy, sex chromosome aneuploidy, and structural abnormality were observed as chromosomal abnormality types. Interestingly, polyploidy and placental mosaicism were not found in this miscarriage group. The incidence of 62.35% may be underestimated as many miscarriages occur very early before a woman realizes she is pregnant. Limitations of the BoBs assay are its ability to detect only genomic imbalances with a certain resolution but not all chromosomal aberrations. This technique cannot detect balanced structural aberrations, ploidy changes, and mosaicism which can only be detected by conventional karyotyping.

Consistent with previous studies, trisomy 16, 22, and 21 were the most common abnormalities in miscarriages [Hassold et al., 1980; Eiben et al., 1990; Ljunger et al., 2005; Soler et al., 2017; Pylyp et al., 2018]. Some particular types of chromosomal abnormalities lead to miscarriage status. By contrast, others such as trisomy 13, 18, 21, and sex chromosomal aneuploidy are compatible with life and found in intrauterine survivals, second-trimester pregnancies, and live-born infants.

Our results showed chromosomal abnormalities in intrauterine survivals at only 10–13^+6^ weeks of gestation, with the highest rate found in 12–13^+6^ weeks of gestation, possibly because CVS is usually carried out between the 11th and 14th week of pregnancy and is only offered if there is a high chance of genetic or chromosomal condition. The frequency of chromosomal abnormalities in miscarriages was not significantly correlated with maternal age ≥35 years (*p* < 0.05). Therefore, other factors apart from age may cause chromosomal nondisjunction in younger women [Ahmad et al., 2010]. A possible explanation for our data involves environmental toxic substance exposure. Economic development and rapid urbanization in Thailand have resulted in environmental degradation and air pollution from traffic in inner cities, chemical hazards from pesticides in countryside areas, heavy metal contaminants from industrial zones and mining, and electronic appliance waste pose a risk to maternal and fetal health [Sinitkul et al., 2018; Waeyeng and Yimthiang, 2021].

The incidence and types of fetal chromosomal abnormalities during first trimester miscarriages in this study concur with previous reports, except for the correlation between chromosomal abnormalities and maternal age ≥35 years.

To the best of our knowledge, no data are available on prenatal chromosomal abnormalities during the first trimester of Thai pregnancies for miscarriages and intrauterine survivals. This may be due to the difficulty of conducting chorionic villus sampling and tissue culture techniques. Obstetricians and technicians must be experienced and possess excellent skills to conduct the medical procedure. Proper handling of first trimester chorionic villus samples in a cytogenetic laboratory requires well-trained cytogeneticists. The genetic characteristics outlined in this study provide vital information for medical management, reproductive counseling, and supportive patient care.

## Statement of Ethics

The study was conducted ethically in accordance with the World Medical Association Declaration of Helsinki. The Ethical Clearance Committee, Faculty of Medicine, Ramathibodi Hospital, Mahidol University approved this study on human rights related to research involving human subjects (COA. MURA2021/811). Patient consent was waived due to using not re-identifiable data from the laboratory results.

## Conflict of Interest Statement

The authors have no conflicts of interest to declare.

## Funding Sources

This study was not specifically funded.

## Author Contributions

Rachanee Parinayok: study design, chromosome analysis, scientific data collection, data analysis, result interpretation, manuscript preparation. Prapatsorn Areesirisuk: genetic testing analysis, critical revision of the manuscript. Takol Chareonsirisuthigul: genetic testing analysis, statistical analysis, revision of the manuscript. Warakorn Buchachat: chromosome analysis. Budsaba Rerkamnuaychoke: study design, critical revision, final approval of the manuscript.

## Data Availability Statement

All data can be found in the article and its online supplementary material.

## Supplementary Material

Supplementary data

## Figures and Tables

**Table 1:** Chromosomal abnormalities according to gestational age and sex

Types of chromosomal abnormalities	Group I (5–7^+6^ weeks)				Group II (8–9^+6^ weeks)				Group III (10–11 ^+6^ weeks)				Group IV (12–13^+6^ weeks)			
	miscarriages		survivals		miscarriages		survivals		miscarriages		survivals		miscarriages		survivals	
	M	F	M	F	M	F	M	F	M	F	M	F	M	F	M	F
Normal (*n* = 130)	3	5			8	16			8	13	7	6	6	2	20	36

Abnormal (*n* = 135)	2	7			19	27			14	17	1	2	5	10	12	19

Single autosomal trisomy																
4						1										
7						1										
10					1											
12					1											
13					1	1			2				2		2	4
14					1											
15		1			2	2				3				1		
16					3	6			6	3				1		
18		1				1					1				6	5
19	1				1								1			
20	1					1										
21		1			2	2			3	2			2	2	4	3
22		2			4	5			3	3						

Double trisomy																
3 and 18						1										
13 and 16						1										
18 and 22						1										

Combined abnormalities																
Trisomy 6 with X0 Trisomy 19 with X0		1				1				1						
Monosomy 19 with X0 Trisomy 21 with X0		1														

Sex chromosome aneuploidy																
45,X 47,XXY					3	3				5		2		5		6
mos 45,X/47,XXX																1

Structural abnormality																
dup(2)(p25.1p25.3)														1		

**Table 2:** Incidence of main chromosomal abnormalities during the first trimester

Types of chromosomal abnormalities	Abnormal cases (*n* = 135)					
	miscarriages (*n* = 101)			survivals (*n* = 34)		
	male (*n* = 41)	female (*n* = 60)	total (%)	male (*n* = 13)	female (*n* = 12)	total (%)
Single autosomal trisomy	38	39	77 (76.24^a^, 57.04^c^)	13	12	25 (73.53^b^, 18.52^c^)
Double aneuploidy						
Double trisomy	−	3	3 (2.97^a^, 2.22^c^)	−	−	−
Combined abnormalities	−	4	4 (3.96^a^, 2.96^c^)			
Sex chromosome aneuploidy	3	13	16 (15.84^a^, 11.85^c^)		9	9 (26.47^b^, 6.67^c^)
Structural abnormality	−	1	1 (0.99^a^, 0.74^c^)			

a The percentage was calculated concerning the total number of abnormal cases of miscarriages.

b The percentage was calculated concerning the total number of abnormal cases of intrauterine survivals.

c The percentage was calculated concerning the total number of abnormal cases in first trimester.

**Table 3:** Chromosomal abnormalities by maternal age

Types of chromosomal abnormalities	Maternal age of miscarriages						Maternal age of intrauterine survivals					
	<25	25–29	30–34	35–40	>40	total	<25	25–29	30–34	35–40	>40	total
Total cases of pregnant women	4	8	33	102	15	162	8	24	36	25	10	103
Single autosomal trisomy	1 (25.00)	2 (25.00)	11 (33.33)	54 (52.94)	9 (60.00)	77 (47.53)	2 (25.00)	2 (8.33)	3 (8.33)	10 (40.00)	8 (80.00)	25 (24.27)
Double trisomy	−	−	−	1 (0.98)	2 (13.33)	3 (1.85)	−	−	−	−	−	−
Combined abnormalities
Trisomy with X0	−	−	1 (3.03)	2 (1.96)	−	3 (1.85)	−	−	−	−	−	−
Monosomy with X0	−	−	−	1 (0.98)	−	1 (0.62)	−	−	−	−	−	−
Sex chromosome aneuploidy
Monosomy X	−	2 (25.00)	4 (12.12)	6 (5.88)	1 (6.67)	13 (8.03)	2 (25.00)	2 (8.33)	2 (5.56)	2 (8.00)	−	8 (7.77)
47,XXY	−	1 (12.50)	1 (3.03)	1 (0.98)	−	3 (1.85)	−	−	−	−	−	−
mos 45,X/47,XXX	−	−	−	−	−	−	−	−	1 (2.78)	−	−	1 (0.97)
Structural abnormality	−	−	1 (3.03)	−	−	1 (0.62)	−	−	−	−	−	−
Total chromosomal abnormalities	1 (25.00)	5 (62.50)	18 (54.55)	65 (63.73)	12 (80.00)	101 (62.35)	4 (50.00)	4 (16.67)	6 (16.67)	12 (48.00)	8 (80.00)	34 (33.01)

Percentages are given in parentheses.

**Table 4:** Correlation of chromosomal abnormalities with maternal age <35 years and ≥35 years

Comparison by age	Miscarriages		Intrauterine survivals		Total	
	abnormal/normal	*p* value	abnormal/normal	*p* value	abnormal/normal	*p* value
Age <35 years	27/18	0.702	14/54	**0.0000**	41/72	**0.0000**
Age ≥35 years	74/43		20/15		93/58	

Bold characters show significant correlation with maternal age (≥35 years).
