# Supplementary material for: Incidence and Types of Fetal Chromosomal Abnormalities in First Trimester of Thai Pregnant Women between Miscarriages and Intrauterine Survivals
Source: Cytogenet Genome Res. 2023 Mar 1;162(7):345–53. doi: 10.1159/000527977 (PMC10273899; doi:10.1159/000527977)
Supplement: Supplementary file 1 — Supplementary data [file cgr-0162-0345-s01.docx]

**Supplementary Table 1.** Incidence of chromosomal abnormalities.

| Types of chromosomal abnormalities | Numbers of abnormal cases during the first trimester (n=135) | | | | | |
| --- | --- | --- | --- | --- | --- | --- |
|  | Miscarriages (n=101) | | | Survivals (n=34) | | |
|  | Male (n=41) | Female (n=60) | total (%) | Male (n=13) | Female (n=12) | total (%) |
| **Single autosomal trisomy** |  |  |  |  |  |  |
| 4 | - | 1 | 1(0.99^a^,0.74^c^) |  | - | - |
| 7 | - | 1 | 1(0.99^a^,0.74^c^) | - | - | - |
| 10 | 1 | - | 1(0.99^a^,0.74^c^) | - | - | - |
| 12 | 1 | - | 1(0.99^a^,0.74^c^) | - | - | - |
| 13 | 5 | 1 | 6(5.94^a^,4.44^c^) | 2 | 4 | 6(17.65^b^, 4.44^c^) |
| 14 | 1 | - | 1(0.99^a^,0.74^c^) | - | - | - |
| 15 | 3 | 6 | 9(8.91^a^,6.67^c^) | - | - | - |
| 16 | 9 | 10 | 19(18.81^a^,14.07^c^) | - | - | - |
| 18 | - | 2 | 2(1.98^a^,1.48^c^) | 7 | 5 | 12(35.29^b^, 8.87^c^) |
| 19 | 3 | - | 3(2.97^a^,2.22^c^) | - | - | - |
| 20 | 1 | 1 | 2(1.98^a^,1.48^c^) | - | - | - |
| 21 | 7 | 7 | 14(13.86^a^,10.37^c^) | 4 | 3 | 7(20.59^b^, 5.19^c^) |
| 22 | 7 | 10 | 17(16.83^a^,12.59^c^) | - | - | - |
| **Total** | 38 | 39 | 77(76.24^a^,57.04^c^) | 13 | 12 | 25(73.53^b^, 18.5^c^) |
| **Double trisomy** |  |  |  |  |  |  |
| 3 and 18 | - | 1 | 1(0.99^a^, 0.74^c^) | - | - | - |
| 13 and 16 | - | 1 | 1(099^a^, 0.74^c^) | - | - | - |
| 18 and 22 | - | 1 | 1(0.99^a^, 0.74^c^) | - | - | - |
| **Total** | - | 3 | 3(2.97^a^, 2.22^c^) | - | - | - |
| **Combined abnormalities** |  |  |  |  |  |  |
| Trisomy 6 with X0 | - | 1 | 1(0.99^a^, 0.74^c^) | - | - | - |
| Trisomy 19 with X0 | - | 1 | 1(0.99^a^, 0.74^c^) | - | - | - |
| Monosomy 19 with X0 | - | 1 | 1(0.99^a^, 0.74^c^) | - | - | - |
| Trisomy 21 with X0 | - | 1 | 1(0.99^a^, 0.74^c^) | - | - | - |
| **Total** |  | 4 | 4(3.96^a^, 2.96^c^) |  |  |  |
| **Sex chromosome aneuploidy** |  |  |  |  |  |  |
| 45,X | - | 13 | 13(12.87^a^,9.63^c^) | - | 8 | 8(23.53^b^,5.93^c^) |
| 47,XXY | 3 | - | 3(2.97^a^,2.22^c^) | - | - | - |
| mos 45,X/47,XXX | - | - | - | - | 1 | 1(2.94^b^,0.74^c^) |
| **Total** | 3 | 13 | 16(15.8^a^,11.9^c^) |  | 9 | 9(26.47^b^,6.67^c^) |
| **Structural abnormality** |  |  |  |  |  |  |
| dup(2)(p25.1 p25.3) | - | 1 | 1(0.99^a^, 0.74^c^) | - | - | - |
| **Total** |  | 1 | 1(0.99^a^, 0.74^c^) |  |  |  |

^a^ : The percentage was calculated concerning the total abnormal cases of miscarriages.

^b^ : The percentage was calculated concerning the total abnormal cases of intra-uterine survivals.

^c^ : The percentage was calculated concerning the total abnormal cases in first trimester.
